# Chemical Composition and In Vitro Bioactive Potential of Dried Honeybee (*Apis mellifera* L.) Podmore as a Candidate Functional Food Ingredient

**DOI:** 10.1002/fsn3.72471

**Published:** 2026-10-08

**Authors:** Marwa Rashad Ali, Esraa Naser Mohamed, Hatem Sharaf El‐Din, Rawaa H. Tlay, Tarek Gamal Abedelmaksoud

**Affiliations:** ^1^ Food Science Department, Faculty of Agriculture Cairo University Giza Egypt; ^2^ Economic Entomology and Pesticides Department, Faculty of Agriculture Cairo University Giza Egypt; ^3^ Food Science Department, Faculty of Agricultural Engineering Damascus University Damascus Syrian Arab Republic

**Keywords:** α‐glucosidase inhibition, antioxidant activity, *Apis mellifera*, bioactive compounds, chitin, chitosan, honeybee podmore, insulin secretion, pancreatic lipase inhibition

## Abstract

Honeybee ( *Apis mellifera* L.) podmore is an underutilized apicultural by‐product that may serve as a source of nutrients and bioactive compounds. This study evaluated the proximate composition and insulin secretion‐modulating potential of dried honeybee podmore. The results showed that podmore contained a high protein level (47.13 g/100 g dry weight) and a considerable lipid content (19.28 g/100 g dry weight). The amino acid profile was balanced, dominated by glutamic acid and aspartic acid, and contained substantial amounts of essential amino acids, particularly leucine, lysine, and valine. HPLC analysis revealed gallic acid (818.06 μg/g) and chlorogenic acid (225.04 μg/g) as the predominant compounds. Podmore extract exhibited notable antioxidant activity and showed concentration‐dependent inhibition of pancreatic lipase and α‐glucosidase, reaching 96.9% and 83.7% inhibition, respectively, at 1000 μg/mL. The extract enhanced insulin secretion in MIN6 pancreatic β cells under both low‐ and high‐glucose conditions, with the highest response observed at 500 μg/mL under high‐glucose conditions. These findings suggest that dried honeybee podmore may be a promising source of nutritional and bioactive compounds with potential applications in the development of functional foods or nutraceuticals. However, further studies, including safety assessment, bioavailability evaluation, and in vivo validation, are required before its practical application.

## Graphical abstract

Dried honeybee podmore was evaluated as a sustainable functional food ingredient. The extract showed high protein, lipids, balanced amino acids, chitin, chitosan, minerals, phenolics, and flavonoids. It exhibited antioxidant activity, inhibited pancreatic lipase and alpha glucosidase, and enhanced insulin secretion in MIN6 beta cells, supporting potential nutraceutical applications after further validation studies.

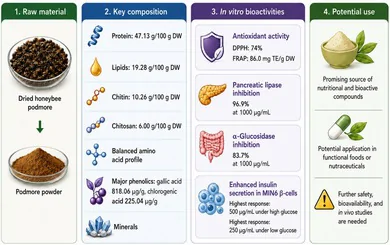

## 1 Introduction

Dietary bioactive compounds have attracted increasing attention in food science and nutrition for their potential to support health and reduce oxidative and metabolic stress. Natural food ingredients rich in proteins, phenolic compounds, polysaccharides, and other bioactive constituents are increasingly being explored for their possible use in functional foods and nutraceutical products (Luo et al. 2021; Chavda et al. 2023). Among natural sources, honeybee products such as honey, propolis, bee pollen, royal jelly, and bee bread have been widely investigated because of their complex chemical composition and reported antioxidant, antimicrobial, anti‐inflammatory, and metabolic effects (Khalifa et al. 2021; El Didamony et al. 2024; Anjos and Miguel 2025).

In addition to conventional bee products, attention has recently increased toward underutilized apicultural byproducts. Honeybee podmore, a material consisting of dried, dead honeybees collected from hives, is an interesting but still poorly explored material. Previous studies have suggested that honeybee‐derived materials may contain valuable nutritional and bioactive components, including proteins, lipids, minerals, chitin, chitosan, phenolic compounds, and other secondary metabolites (Ixtiyarova et al. 2020; Abedelmaksoud et al. 2024). From a sustainability perspective, the valorization of podmore may help reduce apicultural waste while providing a potential source of functional ingredients for food, feed, or nutraceutical applications.

The nutritional relevance of insect‐derived materials is mainly associated with their high protein content, essential amino acids, lipid fraction, minerals, and fiber‐like components such as chitin (Rumpold and Schlüter 2013; Finke 2007). Chitin and its deacetylated derivative, chitosan, have received particular attention because of their potential physiological effects, including interactions with lipid metabolism, glucose regulation, and gut health (Lopez Santamarina et al. 2020; Priyanka et al. 2022). In addition, phenolic compounds and flavonoids present in bee‐related products may contribute to antioxidant activity and enzyme inhibitory effects through radical scavenging, metal chelation, and modulation of digestive enzymes involved in carbohydrate and lipid metabolism (Gülçin 2020; Kashtoh and Baek 2022).

Despite this potential, honeybee podmore remains less studied than other apicultural products such as propolis, pollen, and honey. Most available studies have focused either on the chemical composition of honeybee bodies or on selected biological activities, whereas a comprehensive evaluation of dried honeybee podmore as a potential bioactive food ingredient remains limited (Melo da Cunha et al. 2018; Abedelmaksoud et al. 2024). In particular, limited information is available on the combined assessment of its proximate composition, amino acid profile, mineral content, chitin and chitosan levels, phenolic and flavonoid composition, antioxidant capacity, digestive enzyme inhibitory activity, and insulin‐secretion‐modulating potential.

Research specifically addressing honeybee podmore remains scarce and fragmented. Previous investigations have primarily focused on whole‐bee preparations or on recovering specific fractions from dead honeybees. Draczyński (2008) demonstrated that naturally dead *Apis mellifera* corpses could serve as an alternative source of chitin, whereas Ixtiyarova et al. (2020) subsequently described chemical procedures for obtaining chitin and chitosan from dead honeybees. Regarding biological activity, *A. mellifera* bee tea showed antioxidant, antiglycation, antihyperglycemic, and antidiabetic effects, supporting the functional potential of whole‐bee preparations (Melo da Cunha et al. 2018). Lapynina (2021) further showed that the transfer of selected macro‐ and trace elements into aqueous extracts of honeybee podmore was influenced by pre‐drying, grinding, and extraction temperature. More recently, dead‐bee‐derived chitosan nanoparticles have been investigated as carriers of apitoxin for antimicrobial and cytotoxic applications (Sharaf et al. 2024). Related compositional research on dried *A. mellifera* adults and pupae has also demonstrated marked effects of caste and developmental stage on proximate composition, amino acid profiles, mineral content, phenolic compounds, and antioxidant activity (Abedelmaksoud et al. 2024). Nevertheless, these studies have generally examined individual fractions, preparations, or biological endpoints. An integrated evaluation linking the nutritional and phytochemical composition of honeybee podmore to antioxidant activity, digestive enzyme inhibition, and pancreatic β‐cell function is lacking, providing the rationale for the present study.

Therefore, the present study aimed to evaluate the chemical composition and in vitro bioactive properties of dried honeybee podmore. The study included proximate analysis, amino acid profiling, mineral determination, chitin and chitosan estimation, HPLC profiling of phenolic and flavonoid compounds, antioxidant activity assessment, inhibition of pancreatic lipase and α‐glucosidase, chitinase activity, and insulin secretion response in MIN6 pancreatic β cells. The findings are intended to provide preliminary scientific evidence on the potential value of honeybee podmore as a candidate ingredient for future functional food or nutraceutical applications, whereas recognizing the need for further safety, bioavailability, and in vivo studies before practical use.

## 2 Materials and Methods

### 2.1 Sample Collection and Preparation

Honeybee podmore ( *A. mellifera* L) samples were collected from the apiary of the Faculty of Agriculture, Cairo University, Giza, Egypt, during spring 2024. The apiary was located in a Mediterranean agroclimatic region, with an average temperature of 25°C ± 3°C and a relative humidity of 60%–70%. Bee colonies were maintained under standard apicultural practices to reduce variation associated with environmental and colony‐management factors. The colonies were standardized to similar strength, consisting of seven combs, including five brood combs and two combs containing honey and bee bread, and were headed by sister‐mated queens.

Honeybee podmore, defined as naturally dead adult worker honeybees recovered during routine hive cleaning, was collected from three colonies. Five subsamples were obtained from each colony and pooled on a colony basis to produce three independent composite samples, with each colony representing one biological replicate. Before analysis, the collected honeybees were manually sorted by caste‐specific external morphological characteristics. Worker bees were distinguished by their relatively small body size, dorsally separated compound eyes, tapered abdomen, and the presence of corbiculae (pollen baskets) on the hind tibiae. Individuals exhibiting drone characteristics, particularly a larger body and enlarged compound eyes meeting dorsally, or queen morphology, characterized by a markedly enlarged and elongated abdomen, were excluded (Carreck et al. 2013). Following collection, the samples were immediately stored at −20°C until further processing. Before analysis, samples were dried in a laboratory oven at 40°C for 24 h, milled with an analytical mill, and sieved through a 50‐mesh sieve to obtain a homogeneous powder suitable for subsequent analyses.

### 2.2 Proximate Composition Analysis

The proximate composition of dried honeybee podmore powder, including moisture, crude protein, crude fat, ash, and crude fiber, was determined according to standard AOAC procedures (AOAC 2019). Total carbohydrate was calculated by difference, whereas available carbohydrate was estimated after subtracting crude fiber from total carbohydrate. All results were expressed on a dry weight basis, except moisture, which was expressed as a percentage of the original dried sample.

The energy value was calculated using the Atwater conversion factors according to the following equation:

$$\begin{array}{cc} \text{Energy value}\;\left(\text{kcal}/100\;g\right)\; & =4\times \left(\text{protein}+\text{available carbohydrate}\right)\\ & +9\times \mathrm{fat} \end{array}$$

where protein, available carbohydrate, and fat were expressed as g/100 g sample (FAO 2003; Ganogpichayagrai and Suksaard 2020).

### 2.3 Amino Acid Analysis

The amino acid composition of dried honeybee podmore powder was determined using high‐performance liquid chromatography (HPLC), following the method described by Henderson and Brooks (2010), with appropriate modifications. Briefly, samples were hydrolyzed under acidic conditions, filtered, and prepared for chromatographic analysis. Amino acids were separated using an Agilent 1260 series HPLC system equipped with an Eclipse Plus C18 column, 4.6 × 250 mm, 5 μm. The mobile phase consisted of sodium phosphate‐borate buffer, pH 8.2, as solvent A, and acetonitrile, methanol, and water, 45:45:10, v/v/v, as solvent B. The flow rate was maintained at 1.5 mL/min. Amino acids were identified and quantified by comparison with external amino acid standards analyzed under the same chromatographic conditions. Amino acid contents were reported as mg/g protein.

### 2.4 Mineral Content Analysis

Mineral elements in dried honeybee podmore powder were determined after wet acid digestion according to AOAC procedures (AOAC 2019). Approximately 0.5–1.0 g of dried and ground sample was accurately weighed into a digestion vessel and digested using a mixture of concentrated nitric acid and perchloric acid. The mixture was heated gradually on a digestion block until a clear digest was obtained, indicating complete decomposition of organic matter. The digest was then filtered and diluted to a known volume with distilled water. Calcium, magnesium, potassium, sodium, iron, zinc, and manganese were quantified using atomic absorption spectrophotometry or an equivalent validated elemental analysis system. Phosphorus was determined colorimetrically using the molybdenum blue method at 880 nm. Calibration curves were prepared using certified standard solutions for each mineral. Results were expressed as ppm on a dry weight basis.

### 2.5 Determination of Chitin and Chitosan

Chitin and chitosan contents in dried honeybee podmore powder were estimated based on glucosamine released after acid hydrolysis, following the principles described by Han and Heinonen (2021) and Šulc (2024), with modifications. Samples were first defatted using Soxhlet extraction, followed by deproteinization under alkaline conditions. The resulting material was hydrolyzed using 6 M HCl at approximately 110°C to convert chitin and chitosan polymers into glucosamine monomers. After hydrolysis, the mixture was cooled, neutralized, filtered, and prepared for derivatization.

Glucosamine derivatization was performed using 9‐fluorenylmethoxycarbonyl chloride, FMOC Cl. The derivatized compounds were analyzed by HPLC using a reversed‐phase C18 column. A gradient mobile phase consisting of acetonitrile and water containing 0.1% formic acid was used at a flow rate of 1.0 mL/min, with detection at 265 nm. Quantification was performed using external calibration curves prepared from glucosamine standards. Chitin and chitosan contents were expressed as g/100 g dry weight after applying appropriate conversion factors.

### 2.6 HPLC Analysis of Phenolic and Flavonoid Compounds

Phenolic and flavonoid compounds in dried honeybee podmore were extracted and analyzed by HPLC following Ali et al. (2024), with slight modifications. Briefly, 100 mg of the powdered sample was mixed with 10 mL of methanol in a capped extraction vessel. Ultrasound‐assisted extraction was performed in an ultrasonic bath (Model FSF‐020S; Faithful Instrument Co. Ltd., China) for 45 min at 25°C ± 2°C, using a fixed ultrasonic frequency of 40 kHz and an output power of 120 W. The extract was subsequently centrifuged using a Hettich Universal 320 centrifuge at approximately 3000 × *g* for 7 min at room temperature (25°C ± 2°C). The supernatant was then filtered through a 0.45 μm membrane filter before HPLC injection.

Separation was performed using an Agilent 1200 HPLC system equipped with a C18 reversed‐phase column (4.6 × 250 mm, 5 μm) maintained at 35°C. Solvent A consisted of water and acetic acid, 98:2, v/v, whereas solvent B consisted of acetonitrile and methanol, 50:50, v/v. Gradient elution was applied for 55 min at a flow rate of 1.0 mL/min. Phenolic acids and flavonoids were detected at 280 and 330 nm, respectively. Identification and quantification were carried out by comparison with authentic external standards. Authentic analytical reference standards of gallic acid, chlorogenic acid, methyl gallate, caffeic acid, kaempferol, quercetin, vanillin, hesperetin, pyrocatechol, syringic acid, ellagic acid, naringenin, coumaric acid, daidzein, ferulic acid, cinnamic acid, catechin, rutin, and rosmarinic acid were obtained from Sigma‐Aldrich (St. Louis, MO, USA). Compound identification was performed by comparing sample retention times with those of the corresponding reference standards analyzed under identical chromatographic conditions. Quantification was performed using compound‐specific external calibration curves.

### 2.7 Preparation of Podmore Extract for In Vitro Bioactivity Assays

For in vitro bioactivity assays, dried honeybee podmore powder was extracted using the same methanolic extraction conditions as those used for phenolic profiling. The extract was centrifuged at approximately 3000 × *g* for 7 min at room temperature (approximately 25°C ± 2°C), filtered through a 0.45 μm membrane filter, and aliquots were prepared at the required working concentrations for the antioxidant and enzyme‐inhibitory assays. For the insulin secretion assay, the dried extract residue was reconstituted in Krebs‐Ringer buffer to obtain the tested concentrations, whereas maintaining the same vehicle conditions across treatments and controls.

### 2.8 Antioxidant Activity

#### 2.8.1 DPPH Radical Scavenging Activity

The free radical scavenging activity of honeybee podmore extract was determined using the DPPH assay, as described by Ali et al. (2024), with slight modifications. Briefly, 0.1 mL of extract solution was mixed with 3.9 mL of DPPH solution prepared in methanol. The reaction mixture was incubated in the dark at room temperature for 30 min, and absorbance was measured at 517 nm using a UV–visible spectrophotometer. DPPH radical scavenging activity was calculated as follows:

$$\text{DPPH scavenging activity}\;\left(\%\right)=\left[\left(A_{\text{control}}-A_{\text{sample}}\right)/A_{\text{control}}\right]\times 100$$

where *A*_control_ is the absorbance of the control reaction and *A*_sample_ is the absorbance of the sample reaction. All measurements were performed in triplicate.

#### 2.8.2 Ferric Reducing Antioxidant Power, FRAP

The ferric reducing antioxidant power of honeybee podmore extract was determined as described by Benzie and Strain (1996). The assay was based on the reduction of ferric tripyridyltriazine complex to the ferrous form under acidic conditions. Absorbance was measured spectrophotometrically, and results were expressed as mg Trolox equivalent per g dry weight.

### 2.9 Enzyme Inhibitory Assays

The inhibitory effects of honeybee podmore extract on enzymes related to carbohydrate and lipid digestion were evaluated using α‐glucosidase and pancreatic lipase inhibition assays. All assays were performed at different extract concentrations, and appropriate blanks, negative controls, and positive controls were included to correct for background absorbance and possible color interference from the extract.

#### 2.9.1 α‐Glucosidase Inhibition Assay

The α‐glucosidase inhibitory activity was determined using p‐nitrophenyl α‐D‐glucopyranoside (pNPG) as the substrate, according to a standard colorimetric method with appropriate modifications (Kumar et al. 2011; Kashtoh and Baek 2022). The enzyme solution was pre‐incubated with different concentrations of honeybee podmore extract at 37°C. The reaction was initiated by adding pNPG and stopped using a sodium carbonate solution. The release of p‐nitrophenol was measured at 405 nm. Acarbose was used as the positive control. Percentage inhibition was calculated relative to the control reaction without extract.

#### 2.9.2 Pancreatic Lipase Inhibition Assay

Pancreatic lipase inhibitory activity was evaluated using porcine pancreatic lipase and p‐nitrophenyl palmitate (pNPP) as the substrate, according to Gil Rodríguez and Beresford (2020), with modifications. The enzyme was pre‐incubated with different concentrations of honeybee podmore extract before initiating the reaction with pNPP. Absorbance was measured at 405 nm. Orlistat was used as the positive control. Percentage inhibition was calculated relative to the control reaction without extract.

### 2.10 Chitinase Activity

Chitinase activity in dried honeybee podmore powder was measured using a commercial enzymatic assay kit according to the manufacturer's instructions. The activity was expressed as U/mg protein, based on the change in absorbance per unit time. The presence of chitinase activity was interpreted as a biochemical characteristic of the sample rather than direct evidence of therapeutic activity. Further enzyme purification and characterization would be required to determine its technological or biological relevance.

### 2.11 Insulin Secretion Assay

The effect of honeybee podmore extract on insulin secretion was evaluated using MIN6 pancreatic β cells. Cells were cultured in Dulbecco's modified Eagle's medium (DMEM), supplemented with 10% fetal bovine serum, penicillin, and streptomycin, and maintained at 37°C in a humidified atmosphere containing 5% CO_2_. Cells were seeded in 96‐well plates and allowed to reach a suitable confluence before treatment.

Honeybee podmore extract was prepared in Krebs‐Ringer buffer and tested at concentrations of 62.5, 125, 250, 500, and 1000 μg/mL under low‐glucose (3.3 mM) and high‐glucose (11.1 mM) conditions. After incubation, culture supernatants were collected, and insulin concentration was determined using an appropriate insulin assay kit according to the manufacturer's protocol. Each treatment was performed in triplicate wells and repeated in three independent experiments.

### 2.12 Statistical Analysis

All analyses were performed using at least three independent replicates, and results were expressed as mean ± standard deviation. Statistical analysis was performed using XLSTAT software (version 2014.5.03; Addinsoft, New York, USA). Data were analyzed using one‐way analysis of variance (ANOVA), followed by Duncan's multiple range test. Differences were considered statistically significant at *p* ≤ 0.05.

## 3 Results and Discussion

### 3.1 Chemical Composition

The proximate composition of dried honeybee podmore is presented in Table 1. The results indicated that dried podmore powder contained a high protein level (47.13 g/100 g dry weight) and a considerable lipid content (19.28 g/100 g dry weight). These values are within the general compositional range reported for several edible insects and insect‐derived products, which are commonly characterized by high protein and moderate‐to‐high lipid contents on a dry‐weight basis (Rumpold and Schlüter 2013; Finke 2007). The relatively high protein content suggests that honeybee podmore may be a valuable protein‐rich byproduct. However, further evaluation of protein digestibility and amino acid quality is required before it can be considered a dietary protein ingredient. The total lipid content of dried honeybee podmore (19.28 g/100 g DW) was close to the 20.02% reported for dried drone pupae and higher than the 14.80% reported for worker pupae by Abedelmaksoud et al. (2024), supporting the plausibility of the present value and indicating that honeybee‐derived powders may represent lipid‐rich insect matrices. In a related physiological study, Güneşdoğdu et al. (2024) demonstrated that the abdominal lipid reserves of adult worker bees varied according to dietary protein source, with the highest abdominal lipid content observed in bees receiving a spirulina‐supplemented diet. Although their values, expressed as mg/bee, cannot be compared numerically with the whole‐body dry‐weight lipid percentage determined in the present study, their findings indicate that bee lipid status is strongly influenced by diet and physiological condition. Accordingly, the lipid content of podmore may vary with bee caste, age, nutritional history, season, and colony‐management practices.

**TABLE 1:** Proximate composition, chitin, and chitosan contents of dried honeybee podmore.

Parameter (g/100 g DW)	Content
Moisture	3.51 ± 0.82
Dry matter	96.49 ± 2.41
Crude protein	47.13 ± 2.67
Total lipids	19.28 ± 1.90
Ash	6.45 ± 0.51
Crude fiber	17.61 ± 1.12
Total carbohydrate	23.63 ± 1.34
Available carbohydrate	6.02 ± 0.57
Chitin	10.26 ± 0.41
Chitosan	6.00 ± 0.91
Estimated energy value (kcal/100 g DW)	386.12

*Note:* Values are expressed as mean ± SD, *n* = 3. Total carbohydrate was calculated by difference. Available carbohydrate was estimated by subtracting crude fiber from total carbohydrate. Energy value was calculated using Atwater factors as follows: 4 × protein +4 × available carbohydrate +9 × fat.

Abbreviation: DW, dry weight.

The total carbohydrate content was 23.63 g/100 g dry weight, whereas the available carbohydrate fraction was 6.02 g/100 g dry weight. This difference is mainly attributable to the high crude fiber content of 17.61 g/100 g dry weight. In insect‐based materials, the fiber fraction is largely associated with chitin, an insoluble structural polysaccharide present in the exoskeleton (Finke 2007; Rumpold and Schlüter 2013). Therefore, the carbohydrate profile of dried honeybee podmore should be interpreted carefully, since a considerable proportion is likely represented by non‐digestible or poorly digestible chitinous material rather than readily available carbohydrate.

The ash content of 6.45 g/100 g dry weight indicates that dried honeybee podmore contains an appreciable mineral fraction. This is consistent with previous reports showing that insect‐derived materials can contribute relevant amounts of macro and trace minerals, depending on species, developmental stage, diet, and environmental conditions (Finke 2002; Ghosh et al. 2021). The low moisture content of 3.51% indicates effective drying and may enhance storage stability by reducing the availability of water for microbial growth. However, additional studies on storage stability and microbiological safety are still needed before practical food application.

Dried honeybee podmore also contained measurable amounts of chitin (10.26 g/100 g dry weight) and chitosan (6.00 g/100 g dry weight). These values support the potential of podmore as a source of chitinous biopolymers. Chitin and chitosan have been studied for their possible technological and physiological relevance, including their use as dietary fiber‐like compounds and their interaction with lipid and glucose metabolism (Lopez Santamarina et al. 2020; Priyanka et al. 2022). Nevertheless, their actual nutritional and functional effects depend on molecular weight, degree of deacetylation, solubility, digestibility, and bioavailability, which were not determined in the present study. Therefore, the present data should be considered as preliminary compositional evidence rather than direct proof of physiological efficacy.

Overall, the proximate composition indicates that dried honeybee podmore is a nutrient‐dense apicultural byproduct rich in protein, lipid, minerals, crude fiber, chitin, and chitosan. These findings support its potential valorization as a candidate ingredient for further research in food, feed, or nutraceuticals. However, safety assessment, protein quality evaluation, digestibility testing, and contaminant screening are essential before recommending its use in human nutrition.

### 3.2 Mineral Content

The mineral composition of dried honeybee podmore powder is shown in Table 2. Among the elements analyzed, phosphorus was the most abundant, at 8100 ppm, followed by potassium at 6000 ppm, calcium at 3500 ppm, and magnesium at 1700 ppm. These results indicate that podmore contains substantial levels of essential macroelements, which may contribute to its nutritional value. Similar observations have been reported for honeybee and insect‐derived materials, although mineral concentrations can vary widely according to species, diet, geographical origin, developmental stage, and analytical conditions (Finke 2002; Ghosh et al. 2021).

**TABLE 2:** Mineral composition of dried honeybee podmore.

Mineral element	Content (ppm, dry weight)
Phosphorus (P)	8100.0 ± 6.02
Potassium (K)	6000.0 ± 1.31
Calcium (Ca)	3500.0 ± 4.10
Magnesium (Mg)	1700.0 ± 1.62
Sodium (Na)	4.5 ± 0.62
Iron (Fe)	362.0 ± 0.12
Zinc (Zn)	85.0 ± 0.37
Manganese (Mn)	1.7 ± 0.72

*Note:* Values are expressed as mean ± SD, *n* = 3. Mineral concentrations are presented as ppm on a dry weight basis.

The predominance of phosphorus, potassium, calcium, and magnesium in the present podmore powder is generally consistent with previous evidence that dead honeybees contain nutritionally relevant macro‐ and trace elements. Lapynina (2021) identified potassium, magnesium, calcium, and sodium among the principal elements transferred into aqueous extracts of dead bees. However, direct numerical comparison is inappropriate because that study examined 5% aqueous extracts and demonstrated that mineral transfer varied considerably with extraction temperature and duration, whereas the present study quantified elements directly in dried whole‐bee material.

The relatively high phosphorus and potassium levels may reflect the natural mineral composition of bee tissues and their dietary exposure to pollen and nectar sources. Potassium is an important intracellular electrolyte, whereas phosphorus is a major component of phospholipids and nucleic acids and is involved in energy metabolism. Calcium and magnesium also contribute to the nutritional profile of podmore, although their bioavailability from insect‐based matrices remains to be investigated. Since part of the mineral fraction may be associated with structural components or protein complexes, total mineral content alone does not necessarily reflect nutritional availability.

Regarding trace elements, iron was the most abundant at 362 ppm, followed by zinc at 85 ppm and manganese at 1.7 ppm. Iron and zinc are nutritionally important trace elements, and their presence may enhance the value of dried honeybee podmore as a micronutrient‐containing material. However, these levels should be interpreted with caution because insect and apicultural materials may also accumulate environmental contaminants depending on foraging area, soil composition, botanical origin, and exposure to agrochemicals (Rumpold and Schlüter 2013; Ghosh et al. 2021). Therefore, future studies should include a full safety assessment, particularly heavy metals such as lead, cadmium, arsenic, and mercury, as well as pesticide residues and microbial contaminants.

The sodium level was low at 4.5 ppm compared with the other macro elements. This may be related to the dietary and physiological characteristics of honeybees, which depend primarily on nectar and pollen rather than on salt‐rich sources. The low sodium content could be nutritionally favorable, but this interpretation requires confirmation using replicated samples from different apiaries and seasons.

Overall, the mineral profile suggests that dried honeybee podmore may represent a source of essential minerals, particularly phosphorus, potassium, calcium, magnesium, iron, and zinc. Nevertheless, before considering its use in food applications, mineral bioavailability and safety from contaminants must be evaluated.

### 3.3 Chitin and Chitosan Content

The chitin and chitosan contents of dried honeybee podmore are presented in Table 1. The results showed that dried podmore contained 10.26 g/100 g dry weight of chitin and 6.00 g/100 g dry weight of chitosan. These findings confirm that honeybee podmore is a chitinous apicultural byproduct, which is expected because chitin is a major structural polysaccharide in the insect exoskeleton (Finke 2007; Rumpold and Schlüter 2013). The observed chitin level is therefore consistent with the structural nature of honeybee bodies and supports the potential use of podmore as a source of chitin‐based biopolymers.

The chitin content detected in the present dried podmore (10.26%) supports previous evidence that honeybee corpses represent a potentially useful source of insect chitin. Draczyński (2008) recovered approximately 18% purified chitin from naturally dead honeybee corpses. In contrast, Sharaf et al. (2024) reported a chitin yield of approximately 25% from dead honeybees and subsequently converted the isolated material into chitosan nanoparticles. These values are not directly equivalent to the present result because they represent recovered yields following deproteinization, demineralization, and purification rather than composition measured on the same analytical basis. Differences in the selected body material, pretreatment, extraction conditions, and yield calculation may therefore explain the variation among studies. Despite these methodological differences, the present findings, including chitin and chitosan contents of 10.26% and 6.00%, respectively, further demonstrate the potential of dried honeybee podmore as an alternative source of these value‐added biopolymers.

Chitosan, the partially deacetylated derivative of chitin, has attracted increasing interest in food science, nutrition, and biomedical research because of its functional properties, including fat binding capacity, interaction with bile acids, and possible effects on glucose and lipid metabolism (Lopez Santamarina et al. 2020; Priyanka et al. 2022). Several studies have suggested that chitosan and chitosan derivatives may modulate postprandial glucose response, lipid absorption, and oxidative stress through multiple mechanisms, including inhibition of digestive enzymes, binding of dietary lipids, and improvement of gut‐related metabolic responses (Perumal et al. 2016; Tzeng et al. 2022).

However, the physiological relevance of chitin and chitosan depends strongly on their molecular weight, degree of deacetylation, solubility, viscosity, and digestibility. These parameters were not determined in the present study. Therefore, although the measured chitin and chitosan contents support the functional potential of dried honeybee podmore, they should not be interpreted as direct evidence of antidiabetic or hypolipidemic effects. Further characterization of the extracted chitinous fraction, including degree of deacetylation, molecular weight distribution, and in vitro digestibility, is required to understand its nutritional and technological relevance. Taken together, the presence of chitin and chitosan in dried honeybee podmore may contribute to its value as a candidate functional ingredient. Nevertheless, further safety, bioavailability, and in vivo studies are required before recommending podmore‐derived chitinous materials for human nutrition or metabolic health applications.

### 3.4 Amino Acid Composition of Dried Honeybee Podmore

Amino acid composition of dried honeybee podmore protein is provided in Table 3 and stated in mg/g protein. The results showed a diverse and well‐balanced amino acid composition, with high levels of glutamic acid (115.00 mg/g protein) and aspartic acid (94.86 mg/g protein). At the same time, there were sufficient concentrations of essential amino acids, including leucine (75.09 mg/g protein), lysine (65.07 mg/g protein), and valine (70.13 mg/g protein). Thus, due to the presence of such amino acids, along with the notable amounts of alanine (65.13 mg/g) and arginine (50.57 mg/g), one can speak about the high nutritional significance of honeybee podmore as an alternative protein source. As for insect‐derived products, their characteristic feature is the diversity of amino acid composition, which depends on many factors (species, developmental stage, diet, processing, etc.).

**TABLE 3:** Amino acid profile of dried honeybee podmore protein.

Amino acids	Protein (mg/g)
Aspartic	94.86 ± 1.31
Glutamic	115.00 ± 1.95
Serine	40.91 ± 1.07
Histidine	25.17 ± 0.56
Glycine	50.83 ± 1.15
Threonine	45.50 ± 1.11
Proline	35.51 ± 0.76
Tyrosine	30.78 ± 0.54
Cystine	15.16 ± 0.14
Valine	70.13 ± 0.82
Methionine	19.94 ± 0.27
Phenylalanine	39.59 ± 0.58
Isoleucine	40.34 ± 0.61
Leucine	75.09 ± 0.78
Lysine	65.07 ± 0.80
Alanine	65.13 ± 0.72
Arginine	50.57 ± 0.47

*Note:* Values are expressed as mg/g protein. The amino acid profile was interpreted relative to the protein fraction, and this unit should be used consistently in the Methods, Results, Discussion, and table heading. Values are expressed as mean ± SD of triplicate measurements (*n* = 3).

However, a higher content of glutamic and aspartic acids is more interesting with respect to the sensory properties of podmore protein. This amino acid provides an umami flavor and participates in metabolic processes. Moreover, the protein contained a substantial amount of branched‐chain amino acids (BCAAs), including leucine (75.09 mg/g) and isoleucine (40.34 mg/g), which are essential for muscle protein synthesis and metabolism. On the other hand, the sulfur‐containing amino acids methionine and cystine (19.94 and 15.16 mg/g), which are limiting in most plant protein sources, add nutritional value to podmore. Nevertheless, these acids require special attention regarding fortification (FAO 2011).

Unlike other protein sources, which might lack one essential amino acid, podmore from honeybees was found to contain all essential amino acids. Podmore had sufficient amounts of lysine (65.07 mg/g), a common limiting amino acid in cereal proteins, as well as threonine and phenylalanine. Based on these findings, it appears that podmore protein from honeybees is a complete protein containing all essential amino acids, hence making it a potential sole protein ingredient in foods. However, further analysis should include protein digestibility studies and the determination of the protein's DIAAS.

In summary, dried pollen from honeybees is a potential protein source that can enhance food taste due to its high concentrations of glutamic and aspartic amino acids. Also, pollen from honeybees contained balanced levels of all essential amino acids, especially leucine, lysine, and valine. Thus, it can be concluded that honeybee podmore is a good protein source that requires little protein fortification in food products.

### 3.5 Phenolic and Flavonoid Profile

The phenolic and flavonoid profile of dried honeybee podmore extract is presented in Table 4. HPLC analysis revealed several phenolic acids and flavonoids, with gallic acid as the predominant phenolic at 818.06 μg/g, followed by chlorogenic acid at 225.04 μg/g. Other compounds were detected at lower concentrations, including methyl gallate, caffeic acid, vanillin, quercetin, kaempferol, hesperetin, ellagic acid, syringic acid, coumaric acid, naringenin, daidzein, ferulic acid, and cinnamic acid. This profile indicates that dried honeybee podmore contains a diverse spectrum of low‐molecular‐weight bioactive compounds that may contribute to its antioxidant and enzyme‐inhibitory activities.

**TABLE 4:** Phenolic and flavonoid profile of dried honeybee podmore extract using HPLC.

Compound	Content (μg/g)
Gallic acid	818.06 ± 7.43
Chlorogenic acid	225.04 ± 3.09
Methyl gallate	33.08 ± 1.08
Caffeic acid	21.47 ± 0.98
Kaempferol	13.03 ± 0.33
Quercetin	12.40 ± 0.27
Vanillin	11.41 ± 0.21
Hesperetin	8.96 ± 0.12
Pyrocatechol	5.61 ± 0.18
Syringic acid	4.64 ± 0.09
Ellagic acid	4.38 ± 0.08
Naringenin	3.39 ± 0.03
Coumaric acid	3.36 ± 0.04
Daidzein	1.45 ± 0.02
Ferulic acid	1.40 ± 0.04
Cinnamic acid	1.00 ± 0.05
Catechin	ND
Rutin	ND
Rosmarinic acid	ND

*Note:* ND, not detected under the applied HPLC conditions. Values are expressed as μg/g sample. Compound identification and quantification were performed by comparison with authentic external standards. Values are expressed as mean ± SD of triplicate measurements (*n* = 3).

The predominance of gallic acid and chlorogenic acid is nutritionally and functionally relevant. Gallic acid is a well‐known phenolic acid with strong reducing power and radical‐scavenging capacity, whereas chlorogenic acid has been widely associated with antioxidant activity and modulation of carbohydrate‐ and lipid‐metabolism‐related pathways (Gülçin 2020; Kashtoh and Baek 2022). The presence of flavonoids such as quercetin and kaempferol, even at low concentrations, may further enhance the bioactive potential of podmore extract, as these compounds are commonly reported to contribute to antioxidant defense, enzyme inhibition, and modulation of cellular signaling (Gülçin 2020).

The presence of phenolic compounds in honeybee podmore may be related to several factors, including the bee diet, pollen and nectar sources, plant‐derived residues in the digestive tract, and potential interactions with hive materials. Similar observations have been reported for other bee products, particularly bee pollen, propolis, and bee bread, which contain phenolic acids and flavonoids derived mainly from botanical sources (Khalifa et al. 2021; Chavda et al. 2023; Anjos and Miguel 2025). Edible insect‐based materials have also been reported to contain phenolic compounds, either intrinsically or through dietary and environmental exposure, supporting the possibility that insect‐derived matrices may act as carriers of plant‐related bioactives (Gumul et al. 2023).

However, the detected phenolic profile should be interpreted carefully. The presence of phenolic compounds does not alone confirm biological efficacy, since activity depends on extractability, concentration, chemical stability, digestion, absorption, metabolism, and bioavailability. Therefore, although the high gallic acid and chlorogenic acid contents may partially explain the antioxidant and enzyme‐inhibitory activities observed in the present study, further correlation analysis between individual phenolics and the biological assays would strengthen this interpretation.

Overall, the HPLC profile suggests that dried honeybee podmore extract is a potential source of phenolic and flavonoid compounds, particularly gallic acid and chlorogenic acid. These compounds may contribute to the extract's functional properties, but their biological relevance requires further mechanistic, bioaccessibility, and in vivo validation studies.

### 3.6 Antioxidant Activity

The antioxidant activity of dried honeybee podmore extract was evaluated using DPPH radical scavenging activity and ferric reducing antioxidant power (FRAP). The extract exhibited notable antioxidant capacity, which may be attributed to the presence of phenolic acids, flavonoids, peptides, and other reducing compounds in the podmore matrix. Phenolic compounds can act as antioxidants through hydrogen atom donation, electron transfer, metal chelation, and stabilization of free radicals (Benzie and Strain 1996; Gülçin 2020).

The DPPH assay indicated that honeybee podmore extract had considerable free radical scavenging activity, reaching 74% radical scavenging activity under the applied assay conditions. This result is consistent with the detected phenolic profile, particularly the high levels of gallic acid and chlorogenic acid. Gallic acid is recognized for its strong radical scavenging potential due to its hydroxyl groups, whereas chlorogenic acid can contribute to antioxidant activity through both radical neutralization and reducing capacity (Gülçin 2020). In addition, protein‐derived peptides and chitinous compounds may also contribute to the antioxidant behavior of insect‐derived materials, as previously suggested for edible insect ingredients (Rumpold and Schlüter 2013; Gumul et al. 2023).

The FRAP assay further confirmed the reducing potential of honeybee podmore extract, yielding a value of 86.0 mg Trolox equivalent/g DW under the assay conditions used. Unlike DPPH, which is expressed as a percentage of radical‐scavenging activity, FRAP reflects the ferric‐reducing capacity of the extract and should therefore be reported in Trolox‐equivalent units. This value should be interpreted with consideration of the extraction solvent, calibration curve, dilution factor, and sample basis used in the assay (Benzie and Strain 1996).

The antioxidant activity observed in the present study is directionally consistent with that reported for *A. mellifera* bee tea, which exhibited direct radical‐scavenging activity, reduced oxidative damage in human erythrocytes, and inhibited the formation of advanced glycation end‐products (Melo da Cunha et al. 2018). Direct quantitative comparison is nevertheless limited by differences in sample preparation, tested concentrations, assay protocols, and the units used to express antioxidant capacity.

The antioxidant activity observed in the present study may support the potential use of dried honeybee podmore as a source of antioxidant compounds. Nevertheless, antioxidant activity measured by chemical assays does not directly reflect biological antioxidant effects in humans. The activity may change during digestion, absorption, metabolism, food processing, and storage. Therefore, further studies using simulated gastrointestinal digestion, cellular antioxidant assays, and in vivo models are required better to evaluate the physiological relevance of Podmore's antioxidant properties.

In summary, dried honeybee podmore extract exhibited promising antioxidant potential, probably related to its phenolic compounds, flavonoids, and other reducing constituents. However, the FRAP data should be expressed in standard equivalent units, and further bioaccessibility and biological validation studies are needed before confirming its functional antioxidant value.

### 3.7 In Vitro Enzyme Inhibitory Activity of Honeybee Podmore Extract

The in vitro enzyme inhibitory activity of honeybee podmore extract was evaluated against α‐glucosidase and pancreatic lipase, two enzymes involved in carbohydrate and lipid digestion, respectively. As shown in Table 5, the extract exhibited concentration‐dependent inhibitory effects against both enzymes. The pancreatic lipase inhibition increased from 21.2% at 1.95 μg/mL to 96.9% at 1000 μg/mL, whereas α‐glucosidase inhibition increased from 30.9% to 83.7% over the same concentration range. This concentration‐dependent response suggests the presence of bioactive constituents that interact with digestive enzymes involved in glucose and lipid metabolism.

**TABLE 5:** Concentration‐dependent inhibitory activity of dried honeybee podmore extract against pancreatic lipase and α‐glucosidase.

Podmore extract concentration (μg/mL)	Pancreatic lipase inhibition (%)	α Glucosidase inhibition (%)
1000.00	96.9 ± 0.402ᵃ	83.7 ± 0.302ᵃ
500.00	93.9 ± 0.105ᵇ	78.5 ± 0.697ᵇ
250.00	92.7 ± 0.800ᵇ	73.4 ± 0.231ᶜ
125.00	82.2 ± 0.501ᶜ	67.3 ± 0.100ᵈ
62.50	72.7 ± 0.602ᵈ	61.7 ± 0.134ᵉ
31.25	63.2 ± 0.721ᵉ	55.7 ± 0.272ᶠ
15.62	52.5 ± 0.633ᶠ	49.1 ± 0.432ᵍ
7.81	41.5 ± 0.813ᵍ	43.2 ± 0.675ʰ
3.90	32.5 ± 0.942ʰ	37.5 ± 0.198^i^
1.95	21.2 ± 0.110^i^	30.9 ± 0.764ʲ

*Note:* Values are expressed as mean ± SD, *n* = 3. Different superscript letters within the same column indicate significant differences at *p* ≤ 0.05 according to one‐way ANOVA followed by Duncan's multiple range test. Pancreatic lipase inhibition was evaluated using orlistat as the reference inhibitor, whereas α‐glucosidase inhibition was evaluated using acarbose as the reference inhibitor.

The α‐glucosidase inhibitory activity observed in the present study may be associated with the phenolic and flavonoid compounds detected in the extract, particularly gallic acid, chlorogenic acid, quercetin, and kaempferol. Phenolic compounds have been widely reported to inhibit carbohydrate‐hydrolyzing enzymes through interactions with enzyme active sites, hydrogen bonding, hydrophobic interactions, and conformational changes that reduce catalytic activity (Kumar et al. 2011; Kashtoh and Baek 2022). Similar inhibitory effects have been reported for other bee products, particularly propolis, which contains phenolic acids and flavonoids with inhibitory activity against α‐glucosidase and related digestive enzymes (Uddin et al. 2022).

The pancreatic lipase inhibitory effect was particularly strong at higher extract concentrations, reaching 96.9% inhibition at 1000 μg/mL. Pancreatic lipase is a key enzyme responsible for the hydrolysis and absorption of dietary triglycerides, and its inhibition is commonly used as an in vitro indicator of potential anti‐obesity activity (Birari and Bhutani 2007; Gil Rodríguez and Beresford 2020). The observed lipase inhibition may be related to the combined action of phenolic compounds, chitinous materials, peptides, and other extractable constituents. Similar lipase inhibitory activity has been reported for propolis and bee pollen, supporting the possibility that bee‐derived matrices may contain compounds capable of modulating lipid digestion (Khongkarat et al. 2022; Uddin et al. 2022).

However, these findings should be interpreted cautiously. The results are based on in vitro enzyme assays and cannot be directly translated into physiological effects in humans. Overall, honeybee podmore extract showed promising in vitro inhibitory activity against α‐glucosidase and pancreatic lipase. These results support its potential as a source of enzyme‐modulating bioactive compounds. Still, further mechanistic, digestion, bioavailability, and in vivo studies are required before making any claims related to diabetes or lipid management.

### 3.8 Chitinase Activity

The chitinase activity detected in dried honeybee podmore was 47.21 U/mg protein. Chitinase is a hydrolytic enzyme involved in the degradation of chitin, a major structural polysaccharide in insect exoskeletons and fungal cell walls. The presence of chitinase activity in honeybee podmore may be related to the biological nature of the bee body, residual enzymatic activity, or associated microbial and environmental components.

From a functional perspective, chitinase activity may be relevant for the degradation of chitinous materials and could have potential technological applications in chitin processing, bioconversion, or waste valorization. Previous studies have described chitinases as enzymes with broad biological and biotechnological relevance, particularly in chitin degradation, antifungal defense, and bioprocessing applications (Merzendorfer and Zimoch 2003; Rathore and Gupta 2015).

Nevertheless, the detection of chitinase activity should not be interpreted as direct evidence of therapeutic effects. In the present study, the enzyme was neither purified nor characterized, and its optimum pH, thermal stability, substrate specificity, and biological relevance were not assessed. Therefore, this result should be presented as a preliminary biochemical feature of honeybee podmore and requires further characterization before any technological or health‐related claims can be made.

### 3.9 Insulin Secretion Modulating Activity in MIN6 β Cells

The effect of honeybee podmore extract on insulin secretion was evaluated in MIN6 pancreatic β‐cells under low‐glucose (3.3 mM) and high‐glucose (11.1 mM) conditions. As shown in Table 6, the extract increased insulin secretion under both glucose conditions. Under high‐glucose conditions, insulin secretion increased progressively to 40.73 ng/mL at 500 μg/mL, then declined to 30.45 ng/mL at 1000 μg/mL. Under low glucose conditions, the highest insulin secretion was observed at 250 μg/mL, reaching 14.71 ng/mL, followed by lower values at 500 and 1000 μg/mL.

**TABLE 6:** Effect of dried honeybee podmore extract on insulin secretion in MIN6 pancreatic β cells under low and high glucose conditions.

Glucose concentration (mM)	Podmore concentration (μg/mL)	Insulin (ng/mL)
11.1 (High glucose)	62.5	7.96 ± 0.112ᵉ
	125	12.91 ± 0.198ᵈ
	250	26.54 ± 0.768ᵇ
	500	40.73 ± 0.213ᵃ
	1000	30.45 ± 0.879ᶜ
	Sub‐maximal concentration	500 μg/mL
3.3 (Low glucose)	62.5	5.59 ± 0.176ᵉ
	125	9.40 ± 0.185ᶜ
	250	14.71 ± 0.654ᵃ
	500	11.02 ± 0.123ᵇ
	1000	8.74 ± 0.342ᵈ
	Sub‐maximal concentration	250 μg/mL

*Note:* Values are expressed as mean ± SD, *n* = 3. Different superscript letters within each glucose condition indicate significant differences at *p* ≤ 0.05, determined by one‐way ANOVA followed by Duncan's multiple‐range test.

This response suggests that honeybee podmore extract may contain compounds that modulate insulin secretion in β cells. The observed activity could be associated with phenolic compounds, chitin and chitosan derivatives, peptides, or other extractable bioactive constituents. Previous studies have reported that some bee‐derived materials, including bee pollen polysaccharides and propolis, may influence glucose metabolism, β‐cell function, insulin sensitivity, or insulin‐related responses in experimental models (Alassaf et al. 2021; Yang et al. 2021; Sani et al. 2023). Chitosan and its derivatives have also been discussed for their potential roles in glucose and lipid metabolic regulation, although their effects depend on structure, molecular weight, solubility, and biological availability (Lopez Santamarina et al. 2020; Tzeng et al. 2022; Priyanka et al. 2022).

The highest insulin secretion was observed at 500 μg/mL under high‐glucose conditions and at 250 μg/mL under low‐glucose conditions. The decrease in insulin secretion at 1000 μg/mL should be interpreted cautiously unless supported by cell viability data. The reduction in insulin secretion at the highest concentration, 1000 μg/mL, is an important observation. This decline may indicate a nonlinear dose–response, feedback regulation, or possible cytotoxic effects at high extract concentrations. Therefore, the interpretation of insulin secretion data should be supported by cell viability assays, such as MTT, resazurin, or similar cytotoxicity tests. Without cell viability data, it is not possible to confirm whether the lower insulin secretion at 1000 μg/mL reflects biological regulation or reduced cell health.

Beyond the previously reported antioxidant and antihyperglycemic effects of *A. mellifera* bee tea (Melo da Cunha et al. 2018), the present study demonstrated inhibition of pancreatic lipase and α‐glucosidase, along with enhanced glucose‐stimulated insulin secretion in MIN6 cells. To our knowledge, these three endpoints have not previously been evaluated together using honeybee podmore. Consequently, direct benchmarking against previous podmore studies is currently not possible, and confirmation through standardized comparative, mechanistic, and in vivo investigations is warranted.

It is also important to emphasize that MIN6 cell results represent an in vitro cellular model and do not provide direct evidence of antidiabetic activity in vivo. The extract may undergo chemical changes during digestion, absorption, metabolism, and circulation, and the active constituents responsible for insulin secretion remain unidentified. Therefore, future studies should include cytotoxicity assessment, glucose‐stimulated insulin secretion controls, positive controls such as KCl or known insulin secretagogues, mechanistic markers related to β‐cell signaling, and in vivo validation using appropriate animal models.

Taken together, honeybee podmore extract showed a promising ability to modulate insulin secretion in MIN6 β cells, particularly under high‐glucose conditions. However, these findings should be presented as preliminary cellular evidence rather than proof of antidiabetic efficacy.

## 4 Conclusion

The present study provides preliminary evidence that dried honeybee podmore is a nutrient‐dense apicultural byproduct containing considerable levels of protein, lipids, minerals, crude fiber, chitin, chitosan, and phenolic compounds. The detected amino acids, particularly glutamic acid, aspartic acid, leucine, lysine, and valine, suggest a balanced and nutritionally valuable protein profile. The substantial presence of these essential amino acids, particularly branched‐chain amino acids (BCAAs) and lysine, supports the potential of podmore as a viable protein ingredient. However, to fully validate its status as a complete protein source, further evaluation of protein quality, digestibility, and amino acid scoring (particularly regarding sulfur‐containing amino acids) is recommended.

The presence of chitin, chitosan, gallic acid, chlorogenic acid, and other phenolic compounds may contribute to the observed antioxidant activity, pancreatic lipase inhibition, α‐glucosidase inhibition, and modulation of insulin secretion in MIN6 pancreatic β‐cells. Nevertheless, these findings are based mainly on chemical, enzymatic, and cell‐based assays. They should therefore be interpreted as preliminary in vitro evidence rather than as confirmation of antidiabetic, anti‐obesity, or therapeutic efficacy.

Before practical application as a functional food, feed, or nutraceutical ingredient, further studies are required to assess safety, cytotoxicity, allergenicity, microbial quality, pesticide residues, heavy metals, bioaccessibility, bioavailability, and in vivo efficacy, particularly because insect‐derived materials intended for food or feed applications require careful safety evaluation (FAO 2013; Rumpold and Schlüter 2013). Future work should also calculate IC_50_ values for enzyme inhibition, include suitable positive and negative controls, evaluate cell viability, and standardize podmore collection, drying, extraction, and storage conditions. Also, future studies should include side‐by‐side comparisons of honeybee podmore with established bee‐derived products, including propolis, bee pollen, and bee bread, as well as representative edible‐insect ingredients, using harmonized extraction procedures, concentrations, expression units, and assay conditions. Such standardized comparisons are necessary to establish the relative functional potency and practical value of podmore, as methodological differences among published studies currently preclude reliable direct benchmarking. Multi‐season and multi‐apiary sampling would further strengthen the reliability and applicability of the findings.

## Author Contributions

**Marwa Rashad Ali:** conceptualization, methodology, writing – original draft, writing – review and editing, supervision. **Esraa Naser Mohamed:** methodology, conceptualization, writing – original draft, writing – review and editing, software. **Hatem Sharaf El‐Din:** writing – review and editing, methodology, validation. **Rawaa H. Tlay:** conceptualization, methodology, resources, software, writing – review and editing, writing – original draft. **Tarek Gamal Abedelmaksoud:** writing – original draft, writing – review and editing, methodology, conceptualization, supervision, software.

## Funding

This research received no specific grant from any funding agency in the public, commercial, or not‐for‐profit sectors. The study was conducted with routine institutional research support from the Food Science Department, Faculty of Agriculture, Cairo University. The supporting institution had no role in the study design; collection, analysis, or interpretation of the data; writing of the report; or the decision to submit the report for publication.

## Disclosure

Transparency statement: Marwa Rashad Ali, the lead author and manuscript guarantor, affirms that this manuscript is an honest, accurate, and transparent account of the study being reported; that no important aspects of the study have been omitted; and that any discrepancies from the study as planned, and if relevant, registered, have been explained.

## Conflicts of Interest

The authors declare no conflicts of interest.

## Data Availability

The data that support the findings of this study are available from the corresponding author upon reasonable request. Author approval and data responsibility statement: All authors have read and approved the final version of the manuscript. Marwa Rashad Ali, corresponding author and manuscript guarantor, had full access to all of the data in this study and takes complete responsibility for the integrity of the data and the accuracy of the data analysis.

## References

[fsn372471-bib-0001] Abedelmaksoud, T. G. , M. S. El Masarawy , A. B. Altemimi , M. A. Hesarinejad , A. M. Hussein , and S. S. Smuda . 2024. “Drones and Workers of Honeybee *Apis mellifera* L. Dried Powder: Chemical Composition, Antioxidant, and Anticancer Assessment.” Food Science & Nutrition 12, no. 12: 10357–10369. 10.1002/fsn3.4581 PMC11666825 39723047

[fsn372471-bib-0002] Alassaf, F. A. , M. H. M. Jasim , M. Alfahad , M. E. Qazzaz , M. N. Abed , and I. A. Thanoon . 2021. “Effects of Bee Propolis on FBG, HbA1c, and Insulin Resistance in Healthy Volunteers.” Turkish Journal of Pharmaceutical Sciences 18, no. 4: 405–409. 10.4274/tjps.galenos.2020.50024 PMC8430402 34496480

[fsn372471-bib-0003] Ali, M. R. , H. H. Ibrahim , and A. A. Salah Eldin . 2024. “Unveiling the Chemical Composition, Bioactive Profile and Antioxidant Capacity of Dried Egyptian Jew's Mallow Stems as a Promising Anticancer Agent.” Molecules 29, no. 6: 1377. 10.3390/molecules29061377 PMC10975874 38543013

[fsn372471-bib-0004] Anjos, O. , and M. G. Miguel . 2025. “Unveiling the Chemistry and Bioactivity of Bee Products and Their Derivatives.” Food 14, no. 17: 3058. 10.3390/foods14173058 PMC12428232 40941174

[fsn372471-bib-0005] AOAC . 2019. Official Methods of Analysis. 21st ed. AOAC International.

[fsn372471-bib-0006] Benzie, I. F. F. , and J. J. Strain . 1996. “The Ferric Reducing Ability of Plasma, FRAP, as a Measure of Antioxidant Power: The FRAP Assay.” Analytical Biochemistry 239, no. 1: 70–76. 10.1006/abio.1996.0292 8660627

[fsn372471-bib-0007] Birari, R. B. , and K. K. Bhutani . 2007. “Pancreatic Lipase Inhibitors From Natural Sources: Unexplored Potential.” Drug Discovery Today 12, no. 19–20: 879–889. 10.1016/j.drudis.2007.07.024 17933690

[fsn372471-bib-0008] Carreck, N. L. , M. Andree , C. S. Brent , et al. 2013. “Standard Methods for *Apis mellifera* Anatomy and Dissection.” Journal of Apicultural Research 52, no. 4: 1–40. 10.3896/IBRA.1.52.4.03.

[fsn372471-bib-0009] Chavda, V. P. , A. Z. Chaudhari , D. Teli , P. Balar , and L. Vora . 2023. “Propolis and Their Active Constituents for Chronic Diseases.” Biomedicine 11, no. 2: 259. 10.3390/biomedicines11020259 PMC9953602 36830794

[fsn372471-bib-0010] Draczyński, Z. 2008. “Honeybee Corpses as an Available Source of Chitin.” Journal of Applied Polymer Science 109, no. 3: 1974–1981. 10.1002/app.28356.

[fsn372471-bib-0011] El Didamony, S. E. , H. I. Gouda , M. M. Zidan , and R. I. Amer . 2024. “Bee Products: An Overview of Sources, Biological Activities and Advanced Approaches Used in Apitherapy Application.” Biotechnology Reports 44: e00862. 10.1016/j.btre.2024.e00862 PMC11538619 39507381

[fsn372471-bib-0012] FAO . 2003. Food Energy: Methods of Analysis and Conversion Factors. FAO Food and Nutrition Paper 77. Food and Agriculture Organization of the United Nations.

[fsn372471-bib-0013] FAO . 2011. Dietary Protein Quality Evaluation in Human Nutrition: Report of an FAO Expert Consultation. FAO Food and Nutrition Paper 92. Food and Agriculture Organization of the United Nations. 26369006

[fsn372471-bib-0014] FAO . 2013. Edible Insects: Future Prospects for Food and Feed Security. FAO Forestry Paper No. 171. Food and Agriculture Organization of the United Nations.

[fsn372471-bib-0015] Finke, M. D. 2002. “Complete Nutrient Composition of Commercially Raised Invertebrates Used as Food for Insectivores.” Zoo Biology 21, no. 3: 269–285.

[fsn372471-bib-0016] Finke, M. D. 2007. “Estimate of Chitin in Raw Whole Insects.” Zoo Biology 26, no. 2: 105–115. 10.1002/zoo.20123 19360565

[fsn372471-bib-0017] Ganogpichayagrai, A. , and C. Suksaard . 2020. “Proximate Composition, Vitamin and Mineral Composition, Antioxidant Capacity, and Anticancer Activity of *Acanthopanax trifoliatus* .” Journal of Advanced Pharmaceutical Technology & Research 11, no. 4: 179–183. 10.4103/japtr.JAPTR_61_20 PMC7784940 33425701

[fsn372471-bib-0018] Ghosh, S. , P. Herren , V. B. Meyer Rochow , and C. Jung . 2021. “Nutritional Composition of Honey Bee Drones of Two Subspecies Relative to Their Pupal Developmental Stages.” Insects 12, no. 8: 759. 10.3390/insects12080759 PMC8396957 34442325

[fsn372471-bib-0019] Gil Rodríguez, A. M. , and T. P. Beresford . 2020. “Lipase Inhibitory Activity Assay for Fermented Milk.” MethodsX 7: 100999. 10.1016/j.mex.2020.100999 PMC7397403 32775223

[fsn372471-bib-0020] Gülçin, İ. 2020. “Antioxidants and Antioxidant Methods: An Updated Overview.” Archives of Toxicology 94, no. 3: 651–715. 10.1007/s00204-020-02689-3 32180036

[fsn372471-bib-0021] Gumul, D. , J. Oracz , S. Kowalski , et al. 2023. “Bioactive Compounds and Antioxidant Composition of Nut Bars With Addition of Various Edible Insect Flours.” Molecules 28, no. 8: 3556. 10.3390/molecules28083556 PMC10143157 37110790

[fsn372471-bib-0022] Güneşdoğdu, M. , A. Sarıoğlu‐Bozkurt , A. Şekeroğlu , and S. H. Abacı . 2024. “Changes in Vitellogenin, Abdominal Lipid Content, and Hypopharyngeal Gland Development in Honey Bees Fed Diets With Different Protein Sources.” Insects 15, no. 4: 215. 10.3390/insects15040215. PMC11050231 38667345

[fsn372471-bib-0023] Han, X. , and M. Heinonen . 2021. “Development of Ultra High Performance Liquid Chromatographic and Fluorescent Method for the Analysis of Insect Chitin.” Food Chemistry 334: 127577. 10.1016/j.foodchem.2020.127577 32712490

[fsn372471-bib-0024] Henderson, J. W. , and A. Brooks . 2010. Improved Amino Acid Methods Using Agilent ZORBAX Eclipse Plus C18 Columns for a Variety of Agilent LC Instrumentation and Separation Goals. Agilent Technologies.

[fsn372471-bib-0025] Ixtiyarova, G. A. , D. A. Hazratova , B. N. Umarov , and O. M. Seytnazarova . 2020. “Extraction of Chitosan From Died Honey Bee *Apis mellifera* .” Chemical Technology, Control and Management 2020, no. 2: 15–20. 10.34920/2020.2.15-20.

[fsn372471-bib-0026] Kashtoh, H. , and K. H. Baek . 2022. “Recent Updates on Phytoconstituent Alpha Glucosidase Inhibitors: An Approach Towards the Treatment of Type Two Diabetes.” Plants (Basel) 11, no. 20: 2722. 10.3390/plants11202722 PMC9612090 36297746

[fsn372471-bib-0027] Khalifa, S. A. M. , M. H. Elashal , N. Yosri , et al. 2021. “Bee Pollen: Current Status and Therapeutic Potential.” Nutrients 13, no. 6: 1876. 10.3390/nu13061876 PMC8230257 34072636

[fsn372471-bib-0028] Khongkarat, P. , P. Traiyasut , P. Phuwapraisirisan , and C. Chanchao . 2022. “First Report of Fatty Acids in *Mimosa diplotricha* Bee Pollen With In Vitro Lipase Inhibitory Activity.” PeerJ 10: e12722. 10.7717/peerj.12722 PMC8734463 35036098

[fsn372471-bib-0029] Kumar, S. , S. Narwal , V. Kumar , and O. Prakash . 2011. “α Glucosidase Inhibitors From Plants: A Natural Approach to Treat Diabetes.” Pharmacognosy Reviews 5, no. 9: 19–29. 10.4103/0973-7847.79096 PMC3210010 22096315

[fsn372471-bib-0030] Lapynina, E. P. 2021. “Influence of the Technology of Preparation of Water Extract of Bees on the Content of Separate Mineral Elements.” Herald of Ryazan State Agrotechnological University Named After P. A. Kostychev 13, no. 4: 83–89. 10.36508/RSATU.2021.33.33.010.

[fsn372471-bib-0031] Lopez Santamarina, A. , A. C. Mondragon , A. Lamas , J. M. Miranda , C. M. Franco , and A. Cepeda . 2020. “Animal Origin Prebiotics Based on Chitin: An Alternative for the Future? A Critical Review.” Food 9, no. 6: 782. 10.3390/foods9060782 PMC7353569 32545663

[fsn372471-bib-0032] Luo, X. , Y. Dong , C. Gu , X. Zhang , and H. Ma . 2021. “Processing Technologies for Bee Products: An Overview of Recent Developments and Perspectives.” Frontiers in Nutrition 8: 727,181. 10.3389/fnut.2021.727181 PMC8595947 34805239

[fsn372471-bib-0033] Melo da Cunha, J. D. S. , T. M. Alfredo , J. M. Dos Santos , et al. 2018. “Antioxidant, Antihyperglycemic, and Antidiabetic Activity of *Apis mellifera* Bee Tea.” PLoS One 13, no. 6: e0197071. 10.1371/journal.pone.0197071. PMC5988306 29870561

[fsn372471-bib-0034] Merzendorfer, H. , and L. Zimoch . 2003. “Chitin Metabolism in Insects: Structure, Function and Regulation of Chitin Synthases and Chitinases.” Journal of Experimental Biology 206, no. 24: 4393–4412. 10.1242/jeb.00709. 14610026

[fsn372471-bib-0035] Perumal, V. , T. Manickam , K. S. Bang , P. Velmurugan , and B. T. Oh . 2016. “Antidiabetic Potential of Bioactive Molecules Coated Chitosan Nanoparticles in Experimental Rats.” International Journal of Biological Macromolecules 92: 63–69. 10.1016/j.ijbiomac.2016.07.006. 27381582

[fsn372471-bib-0036] Priyanka, D. N. , K. V. Harish Prashanth , and R. N. Tharanathan . 2022. “A Review on Potential Anti‐Diabetic Mechanisms of Chitosan and Its Derivatives.” Carbohydrate Polymer Technologies and Applications 3: 100,188. 10.1016/j.carpta.2022.100188.

[fsn372471-bib-0037] Rathore, A. S. , and R. D. Gupta . 2015. “Chitinases From Bacteria to Human: Properties, Applications, and Future Perspectives.” Enzyme Research 2015: 791,907. 10.1155/2015/791907. PMC4668315 26664744

[fsn372471-bib-0038] Rumpold, B. A. , and O. K. Schlüter . 2013. “Nutritional Composition and Safety Aspects of Edible Insects.” Molecular Nutrition & Food Research 57, no. 5: 802–823. 10.1002/mnfr.201200735. 23471778

[fsn372471-bib-0039] Sani, L. , N. Cardinault , J. Astier , P. Darmon , and J. F. Landrier . 2023. “Poplar Propolis Improves Insulin Homeostasis in Non Diabetic Insulin Resistant Volunteers With Obesity: A Crossover Randomized Controlled Trial.” Antioxidants 12, no. 8: 1481. 10.3390/antiox12081481 PMC10451960 37627476

[fsn372471-bib-0040] Sharaf, M. , A. A. Zahra , M. Alharbi , et al. 2024. “Bee Chitosan Nanoparticles Loaded With Apitoxin as a Novel Approach to Eradication of Common Human Bacterial, Fungal Pathogens and Treating Cancer.” Frontiers in Microbiology 15: 1,345,478. 10.3389/fmicb.2024.1345478. PMC10978808 38559346

[fsn372471-bib-0041] Šulc, M. 2024. “Chitin Quantitation (As Glucosamine) in Food Raw Materials by HPLC‐C18‐DAD With Off‐Line Derivatization.” MethodsX 12: 102,729. 10.1016/j.mex.2024.102729. PMC11066474 38707216

[fsn372471-bib-0042] Tzeng, H. P. , S. H. Liu , and M. T. Chiang . 2022. “Antidiabetic Properties of Chitosan and Its Derivatives.” Marine Drugs 20, no. 12: 784. 10.3390/md20120784 PMC9782916 36547931

[fsn372471-bib-0043] Uddin, S. , P. R. Brooks , and T. D. Tran . 2022. “Chemical Characterization, α‐Glucosidase, α‐Amylase and Lipase Inhibitory Properties of the Australian Honey Bee Propolis.” Foods 11, no. 13: 1964. 10.3390/foods11131964. PMC9266216 35804780

[fsn372471-bib-0044] Yang, S. , Y. Qu , J. Chen , et al. 2021. “Bee Pollen Polysaccharide From *Rosa rugosa* Thunb. Promotes Pancreatic β‐Cell Proliferation and Insulin Secretion.” Frontiers in Pharmacology 12: 688,073. 10.3389/fphar.2021.688073 PMC8273306 34262457

